# Book Reviews

**Published:** 1874-11-01

**Authors:** 


### Book Reviews.

SURGICAL EMERGENCIES: TOGETHER WITH THE EMERGENCIES ATTENDANT ON PARTURITION AND THE TREATMENT OF POISONING. A Manual for the use of General Practitioners. By William Paul Swain, F. R. C. S., Surgeon to the Royal Albert Hospital, Devonport. With eighty-two illustrations. Philadelphia: Lindsay & Blakiston, 1874. Pages, 189.

This compendious little manual can hardly fail to meet with favor among those for whom it was written, viz.: general practitioners. For, although it is mainly a compilation from the works of the best and most recent surgical authors, yet its condensation of what is of practical value in each, will supply the need of those who are summoned to cases of every sort of emergency.

The chapter on antiseptic dressings will be perused with interest by many, since it was written by Dr. Bishop at the request of Prof. Lister. While there are probably few in this country who consider the extraordinary precautions remmended by Prof. Lister as essential to a complete antiseptic dressing, it is undoubtedly well to err rather on the side of caution than of carelessness. Specific directions are also here given as to the use of the aspirator and Esmarch's bandage in bloodless operations.

The work is well written, and everything is sacrificed to conciseness that is not essential to the meaning. Many of the wood cuts are familiar to the profession, as they have appeared in the works of Fergusson, Bryant and Heath, but they are the better for general circulation and all are fairly executed.

THE PHYSIOLOGY OF MAN. By Austin Flint, Jr., M. D. New York: D. Appleton & Co. Chicago: Jansen, McClurg & Co.

This fifth volume completes Prof. Flint's very elaborate treatise on Human Physiology.

The work, as a whole, is one of which the American profession may well feel proud. A full and faithful exponent of the physiological science of our day, it represents not a compilation merely, but the results of a vast amount of original research and experimentation carried on by Prof. Flint during the past eleven years.

In this last volume the special senses and generation are considered.

---

THE PHILOSOPHY OF SPIRITUALISM AND THE PATHOLOGY AND TREATMENT OF MEDIOMANIA. By F. R. Marvin, M. D., Prof. of Psychological Medicine and Medical Jurisprudence in the New York Free Medical College for Women. New York: Asaka K. Butts, Publisher. 12mo.; 68 pages.

This little volume is in the form of two lectures, written, the author tells us, to save those who are about to be drawn into the meshes of spiritualism. He regards spiritualism as the worst form of materialism—materialism of materialism. His first lecture is devoted to clear, concise and convincing arguments against spiritualism, arguments which prove one of two things to be true, namely: spirits are material or spiritualism is all fallacy. The second lecture comprises a description of the pathology and treatment of mediomania. He here considers mediums as suffering under a form of insanity, its causes being predisposing and exciting. It may be idiopathic, but generally sympathetic.

